# Limited SHIV *env* diversification in macaques failing oral antiretroviral pre-exposure prophylaxis

**DOI:** 10.1186/1742-4690-9-40

**Published:** 2012-05-09

**Authors:** Qi Zheng, Susan Ruone, William M Switzer, Walid Heneine, J Gerardo García-Lerma

**Affiliations:** 1 Laboratory Branch, Division of HIV/AIDS Prevention, National Center for HIV, Hepatitis, STD, and Prevention, Centers for Disease Control and Prevention, Atlanta, GA, USA

**Keywords:** Single genome amplification, Pre-exposure prophylaxis, Emtricitabine, Truvada

## Abstract

**Background:**

Pre-exposure prophylaxis (PrEP) with daily Truvada [a combination of emtricitabine (FTC) and tenofovir disoproxil fumarate (TDF)] is a novel HIV prevention strategy recently found to prevent HIV transmission among men who have sex with men and heterosexual couples. Acute infection in adherent persons who fail PrEP will inevitably occur under concurrent antiretroviral therapy, thus raising questions regarding the potential impact of PrEP on early viral dynamics. We investigated viral evolution dynamics in a macaque model of PrEP consisting of repeated rectal exposures to SHIV_162P3_ in the presence of PrEP.

**Results:**

Four macaques were infected during daily or intermittent PrEP with FTC or FTC/TDF, and five were untreated controls. SHIV *env* sequence evolution was monitored by single genome amplification with phylogenetic and sequence analysis. Mean nucleotide divergence from transmitted founder viruses calculated 17 weeks (range = 12–20) post peak viremia was significantly lower in PrEP failures than in control animals (7.2 × 10^-3^ compared to 1.6 × 10^-2^ nucleotide substitutions per site per year, respectively, p < 0.0001). Mean virus diversity was also lower in PrEP failures after 17 weeks (0.13% vs. 0.53% in controls, p < 0.0001).

**Conclusions:**

Our results in a macaque model of acute HIV infection suggest that infection during PrEP limits early virus evolution likely because of a direct antiviral effect of PrEP and/or reduced target cell availability. Reduced virus diversification during early infection might enhance immune control by slowing the selection of escape mutants.

## Background

Oral administration of antiretroviral (ARV) drugs before exposure to HIV (pre-exposure prophylaxis [PrEP]) is a novel prevention strategy to protect high-risk HIV-1-negative people from becoming infected [1]. Two recently completed trials with daily Truvada (a combination of emtricitabine [FTC] and tenofovir disoproxil fumarate [TDF]) have provided the first indication that oral PrEP reduces HIV transmission [2,3]. In these trials, the incidence of HIV-1 was reduced by 44% among men who have sex with men (MSM) and 62.6% among heterosexual couples; efficacy was substantially higher for study participants who reported high adherence or were on study medication [2,3]. An additional clinical trial among serodiscordant couples recently discontinued the placebo arm due to the strong HIV prevention effect seen by TDF (62% fewer infections) and Truvada (73% fewer infections) [4]. A fourth trial (FEM-PrEP) with Truvada among high-risk women was recently stopped due to futility as a result of low adherence.

Since current PrEP regimens are not 100% protective, it is essential to understand the characteristics of acute infections that occur during PrEP use in humans. In contrast to natural HIV infections that are characterized by the absence of selective pressures prior to the development of acquired immune responses, early replication and systemic dissemination during infections that occur during PrEP can face an increased genetic bottleneck due to ARV drug activity. Strong drug selective pressures have the potential to shape early virus evolution and diversification from transmitted/founder viruses and alter host immune responses. Infection of non-human primates with SHIV can be used to model how PrEP influences early viral and immunological parameters. This model can recapitulate human infection by using R5-tropic isolates and low challenge doses that better mimic the transmission dynamics of HIV infection [5]. We recently found that macaques infected with SHIV_162P3_ during prophylactic treatment with FTC or Truvada had lower peak viremias and a more rapid decline of virus loads to set-point levels compared to untreated animals, likely due to continued antiretroviral drug treatment during acute infection [6-8]. Interestingly, macaques infected during PrEP had altered acute immune parameters including delayed maturation of antibody avidity against multiple structural and functional antigens, reduced inflammatory responses, and minimal early CD4+ T cell losses [6-10]. While these unique immune parameters likely reflected reduced virus replication under PrEP, they also raised questions about the dynamics of the virus population during acute infection.

The study of early HIV and SIV sequences by single-genome amplification (SGA) provides an accurate picture of the characteristics of transmitted/founder HIV and SIV variants and the distribution of viral quasispecies during primary infection [11,12]. In the absence of adaptive immune pressure or other selective pressures, virus diversification shortly after infection follows a pattern of random evolution with an almost star like phylogeny and a Poisson distribution of nucleotide substitutions [13]. Here we use SGA and sequence analysis to assess the impact of antiretroviral drugs during PrEP failure on viral dynamics. We demonstrate that PrEP confers a severe bottleneck that significantly slows early virus evolution and diversification. These findings in macaques highlight the need to better understand the impact of PrEP on acute HIV infection and the natural course of disease in humans.

## Results

### Dynamics of acute viral infection in treated and untreated macaques

**Figure 1:**
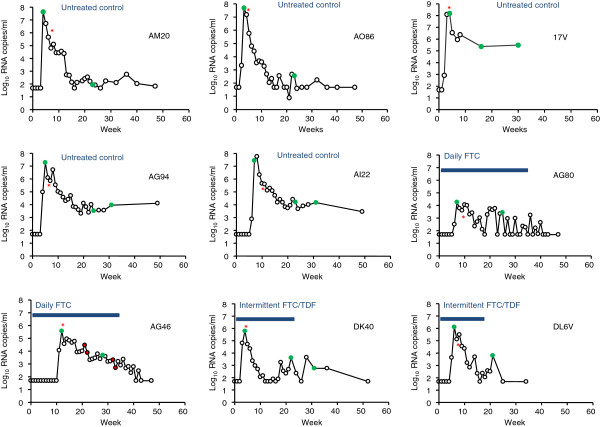
**Viral kinetics and serologic responses in acute SHIV**_**162P3**_**infections.** Macaques AM20, AO86, 17 V, AG94, and AI22 are untreated controls. Macaques AG80, AG46, DK40, and DL6V were infected during concurrent PrEP. All 9 macaques were exposed rectally once a week until the first evidence of SHIV RNA in plasma. Red asterisks indicate seroconversion and green circles indicate time points that were analyzed by SGA. The 4 red dots in macaque AG46 represent time points with detectable M184V mutation (8). Horizontal lines denote the period of time under ARV drug exposure.

Figure 1 shows the kinetics of virus replication and seroconversion in the nine treated and untreated SHIV-infected macaques. Peak viremias in untreated control macaques AG94, AI22, AO86, AM20, and 17 V were 7.3, 7.8, 7.7, 7.6, and 8.2 log_10_ RNA copies/ml, respectively. Peak viremias in the two daily FTC (4.3 and 5.6 log_10_ copies/ml) and the two intermittent Truvada failures (5.8 and 6.1 log_10_ copies/ml) were lower, consistent with the generally blunted acute viremias seen in macaques infected during PrEP in previous studies [6-8] (Figure 1). Macaques AG80 and AG46 received daily FTC for 29 and 24 weeks after the first detectable SHIV RNA in plasma, respectively; AG46 developed the M184V mutation associated with FTC resistance 11 weeks after infection (Figure 1; red dots). The frequency of M184V-containing mutants determined by sensitive real-time PCR was low (16-20%) at week 11, below the assay cutoff (0.6%) at week 12, and high (100%) 22 weeks after infection (not shown) [8]. Macaques DK40 and DL6V also continued receiving two weekly doses of Truvada for 19 and 14 weeks after infection, respectively (Figure 1). Neither of these two macaques had detectable mutations associated with FTC (M184V) or tenofovir (TFV) (K65R) resistance (not shown), despite the long intracellular half-life of FTC-triphosphate and TFV-diphosphate [7,8]. All animals seroconverted within 2–5 weeks after the first detection of RNA in plasma (Figure 1).

### Viral diversity in the SHIV_162P3_ virus stock and in control untreated SHIV_162P3_ infections

**Figure 2:**
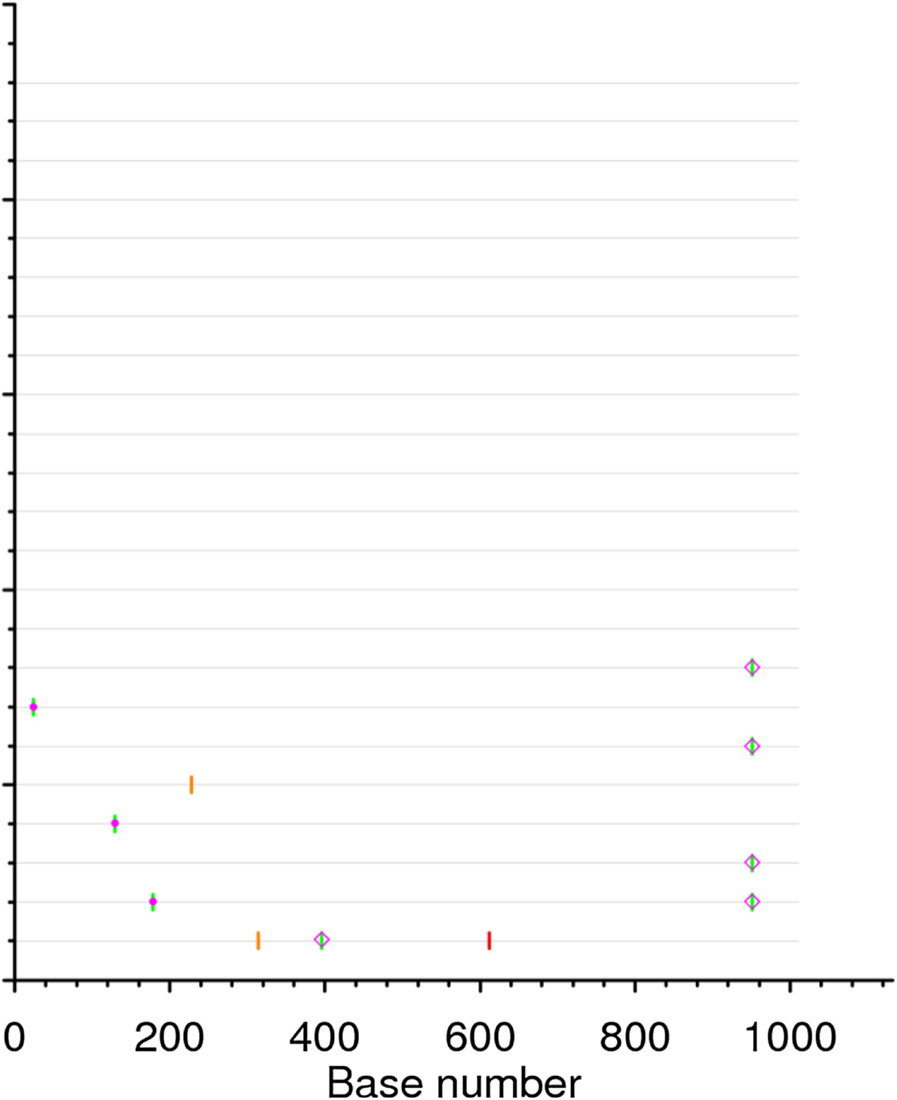
**Highlighter plot of*env*sequences generated from the SHIV**_**162P3**_**challenge stock by single genome amplification.** Nucleotide changes are colored as follows: A, green; T, red; G, orange; C, light blue. Gaps are indicated in gray. Circles denote APOBEC-mediated G-to-A hyper mutation. Diamonds represent G-to-A conversions.

Twenty-three *env* nucleotide sequences were generated by SGA for the SHIV_162P3_ stock. Consistent with the limited heterogeneity of the SHIV_162P3_ virus stock [14,15], the overall nucleotide diversity was found to be low (mean = 0.09%; min, max = 0–0.5%) (Figure 2).

**Figure 3:**
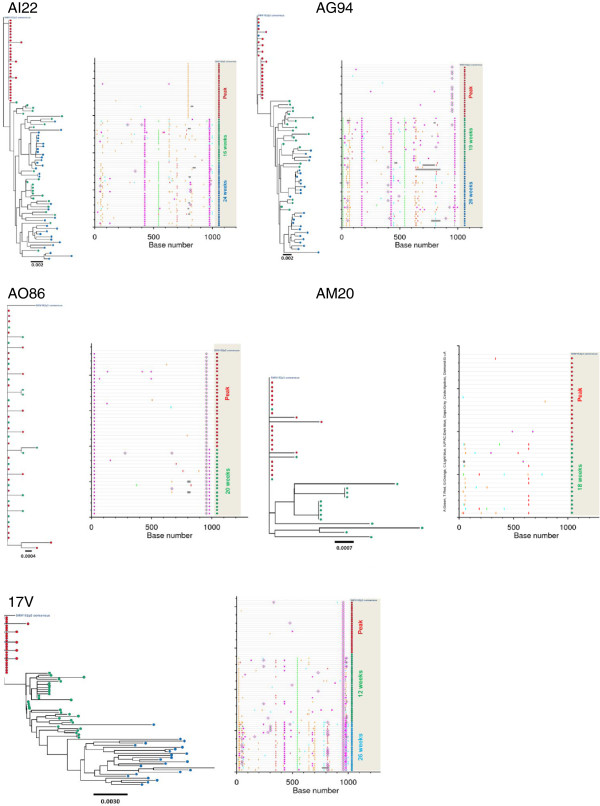
**Neighbor-joining tree and highlighter analysis of*env*sequences generated by single genome amplification in untreated control macaques AI22, AO86, AG94, AM20, and 17 V.** Nucleotide changes are colored as follows: A, green; T, red; G, orange; C, light blue. Gaps are indicated in gray. Circles denote APOBEC-mediated G-to-A hyper mutation. Diamonds represent G-to-A conversions.

We generated 326 *env* sequences from plasma by SGA in the five untreated control macaques infected rectally with SHIV_162P3_. The specific time points used for SGA analysis are denoted as green dots in Figure 1 and are referred as weeks after peak viremia. Of the 326 env sequences, 88 were obtained from macaque AI22 (30 at peak viremia, 24 after 16 weeks, and 34 after 24 weeks), 72 from macaque AG94 (23 at peak viremia, 21 after 19 weeks, and 28 after 26 weeks), 48 from macaque AO86 (28 at peak viremia and 20 after 20 weeks), 37 from macaque AM20 (20 at peak viremia and 17 after 18 weeks), and 81 from macaque 17 V (27 at peak viremia, 31 at week 12, and 25 at week 25). Figure 3 shows the neighbor-joining tree and Highlighter plots generated with *env* sequences from these macaques and demonstrate that all 5 animals were infected by a single variant as indicated by the limited number of *env* sequences seen at peak viremia [16].

**Figure 4:**
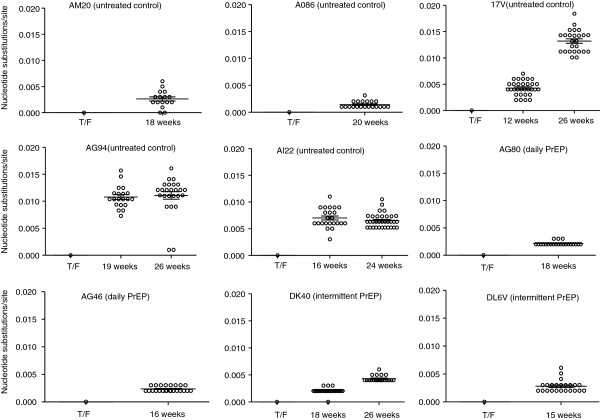
**Viral divergence from the transmitted founder (T/F)*env*sequence in SHIV**_**162P3**_**-infected untreated controls (AM20, AO86, 17 V, AG94, and AI22) and PrEP-treated macaques (AG80, AG46, DK40, and DL6V).** V1-V5 env sequences were amplified by single genome amplification from longitudinal plasma specimens collected 15 to 26 weeks after peak viremia. For the analysis of divergence, nucleotide sequences were aligned and the means and standard deviations for all pair-wise comparisons between individual sequences and the T/F were calculated using the maximum composite likelihood in MEGA5.

We next calculated the genetic diversity of *env* sequences obtained from longitudinal plasma specimens collected at several time points after infection. Mean *env* diversity in control macaque AI22 at peak viremia was 0.02% (min, max = 0–0.3) and increased to 0.72% after 16 (min, max = 0–1.6) and 24 weeks (min, max =0-1.7). Similarly, mean *env* diversity in control macaque AG94 was 0.1% (min, max = 0–0.40) at peak viremia, 0.92% (min, max = 0.1-1.9) after 19 weeks, and 0.96% (min, max = 0–1.9) after 26 weeks. For macaques AO86, AM20, and 17 V, mean env diversity at peak viremia was 0.07% (min, max = 0, 0.4), 0.04% (min, max = 0, 0.3) and 0.04% (min, max = 0, 0.3), respectively. Diversity increased in macaques AM20 (mean = 0.43%; min, max = 0–1.0) and 17 V (mean = 0.47; min, max = 0, 1.1) after 18 and 12 weeks, respectively, and remained low in macaque A086 after 20 weeks (mean = 0,08; min, max = 0–0.3). Figure 4 shows the increasing *env* nucleotide divergence from transmitted founder viruses seen in 4 of the 5 control macaques over time. Mean nucleotide substitutions per site per year in these macaques were 1.6 x 10^-2^ and ranged from 3.7 × 10^-3^ in AO86 to 3.0 × 10^-2^ in AG94.

### Limited viral diversity in SHIV_162P3_-infected macaques failing daily or intermittent PrEP with FTC or Truvada

**Figure 5:**
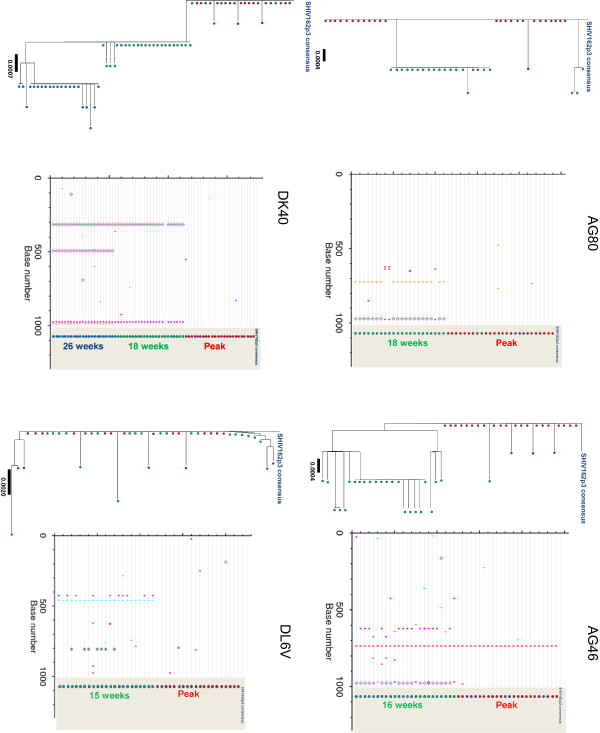
**Neighbor-joining and highlighter analysis of*env*sequences generated by single genome amplification in PrEP breakthrough infections.** Animals AG80 and AG46 were infected during daily PrEP with FTC, and animals DK40 and DL6V were infected during intermittent PrEP with Truvada. Nucleotide changes are colored as follows: A, green; T, red; G, orange; C, light blue. Gaps are indicated in gray. Circles denote APOBEC-mediated G-to-A hyper mutation. Diamonds represent G-to-A conversions.

We next explored the degree of viral diversity in the four macaques infected with SHIV_162P3_ during daily or intermittent PrEP. Figure 5 shows the neighbor-joining tree and Highlighter plots in these four PrEP breakthrough animals showing the limited viral *env* diversity in these animals. Mean *env* nucleotide diversity in macaques AG80 and AG46 that failed daily FTC was low both at peak viremia and after 16 to 18 weeks of follow up. In these two animals, the overall *env* diversity at peak viremia was 0.015% (min, max = 0, 0.2) and 0.025% (min, max = 0, 0.2), respectively. Virus diversity remained low in AG80 at week 18 (mean = 0.12; min, max = 0, 0.6) and in AG46 at week 16 (mean = 0.26; min, max = 0, 0.6) (Figure 5).

Low diversity was also seen in macaques DL6V and DK40 that failed intermittent PrEP with two weekly doses of Truvada. Diversity of *env* sequences at peak viremia was 0.05% (min, max = 0, 0.2) and 0.025% (min, max = 0, 0.2) for macaques DL6V and DK40, respectively, and remained low at week 15 in DL6V (mean = 0.12; min, max = 0, 0.5), and at weeks 18 and 26 in DK40 (mean = 0.04 (min, max = 0, 0.3) and 0.11 (min, max = 0, 0.5), respectively).

We also evaluated *env* nucleotide divergence from the transmitted founder viruses after 17 weeks in all 4 animals infected during PrEP (Figure 4). Mean nucleotide substitutions per site per year were 6.2 × 10^-3^ (min, max = 5.8 × 10^-3^, 8.7 × 10^-3^) for AG80, 7.6 × 10^-3^ (min, max = 3.2 × 10^-3^, 9.8 × 10^-3^) for AG46, 5.9 × 10^-3^ (min, max = 0, 1.2 × 10^-2^) for DK40, and 9.7 × 10^-3^ (min, max = 3.5 × 10^-3^, 1.8 × 10^-2^) for DL6V.

**Figure 6:**
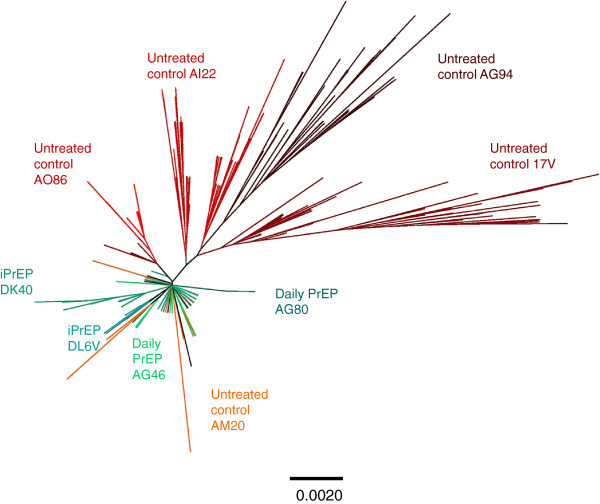
**Phylogenetic analysis of SHIV**_**162P3**_**challenge stock and longitudinal*env*sequences generated by single genome amplification in all six infected macaques.** The neighbor-joining method was used for the analysis. V1-V5 *env* sequences from individual macaques are color coded. PrEP breakthrough infections are characterized by a reduced generation of virus diversity relative to untreated animals as evidenced by the length of the branches.

The degree of *env* diversity and nucleotide divergence from the transmitted founder was compared between PrEP breakthrough and untreated control animals. To normalize for a similar follow up time, this analysis was limited to the first 17 weeks of infection (range = 12–20 for controls and 15–18 for PrEP breakthroughs), and excluded the late time points that were only available in a few animals (24 weeks for AI22, and 26 weeks for AG94, 17 V, and DK40). Over this 17 week period of time, the overall nucleotide divergence from transmitted founder viruses in the 4 PrEP breakthrough animals (7.2 × 10^-3^; min, max = 5.9 × 10^–3^, 9.7 × 10^-3^) was significantly lower than in the five untreated control animals (1.6 × 10^-2^; min, max = 3.7 × 10^-3^, 3.0 × 10^-2^, p < 0.0001) (Figure 4). Table 1 shows that the overall env diversity in the 4 PrEP breakthrough infections was also significantly lower than that seen in the 5 untreated control macaques both at peak viremia and after 17 weeks of infection. The Neighbor-joining phylogenetic analysis of all 9 macaques and the SHIV_162P3_ virus stock *env* sequences are shown in Figure 6 and illustrate how PrEP blunts the initial generation of virus diversity and diversification relative to untreated control animals.

**Table 1:** **SHIV**_**162P3**_***env*diversity in PrEP breakthroughs and untreated control macaques**

	Mean nucleotide diversity (%) (range)		
	**PrEP**	**Controls**	**P value**
Peak	0.027 (0.015-0.05)	0.053 (0.02-0.10)	P < 0.0001
Week 17*	0.14 (0.04-0.26)	0.53 (0.08-0.92)	P < 0.0001

* Sequences were obtained an average of 17 weeks post peak viremia for the 4 PrEP breakthroughs (15, 16, 18, and 18 weeks) and the 5 untreated controls (12, 16, 18, 19, and 20 weeks).

### Amino acid mutations in variable regions of Env gp120

**Figure 7:**
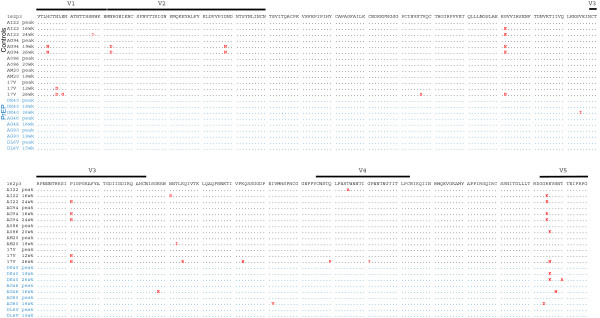
**Consensus Env V1-V5 amino acid sequences generated by single genome amplification in longitudinal specimens from untreated and PrEP breakthrough animals.** Sequences are aligned with a reference SHIV_162P3_ isolate (GenBank accession number AF536757). Amino acid changes in gp120 are shown for each animal.

To evaluate the effect of the observed nucleotide substitutions on Env protein evolution, we examined amino acid changes in the V1-V5 regions of consensus *env* sequences from all 9 macaques. No changes were observed in V1-V4 Env sequences from the four PrEP breakthroughs animals during the 15–26 weeks of follow up (Figure 7). Macaque DK40 had a single V451K change in V5, and macaque AG46 had a novel potential N-linked glycosylation site at position 453 of V5 (Figure 7). In contrast, 4 of the 5 untreated control macaques acquired multiple amino acid changes overtime in V1, V2, V3, or V5. At weeks 19 and 26 after peak viremia, untreated macaque AG94 acquired a novel potential N-linked glycosylation site at position 129 (H129N) in V1, N148D and D184N changes in V2, a P296H change in V3, and an R420K change in V5. Similarly, untreated macaque AI22 acquired the P296H change in V3 and the R420K/E change in V5 over time; macaque AO86 acquired an E421K change in V5, and macaque 17 V acquired N132D in V1, P296H in V3, and E421N in V5 (Figure 7). The Geno2pheno algorithm did not predict a change in co-receptor tropism associated with P296H in V3. Control macaque AM20 did not acquire any amino acid changes in V1-V5 during this period of time (Figure 7).

## Discussion

We used a macaque model of rectal SHIV transmission to explore the impact of PrEP on early virus evolution. Previously, we had found that SHIV infection of macaques during concurrent PrEP was characterized by 100- to 1000-fold lower peak viremias and a more rapid virus decline compared to untreated macaques [6-8]. It is unlikely that these low peak viremias are due to specific MHC-I genotypes since MHC-I alleles do not affect virus loads within the first few weeks of infection [17]. While MHC-I alleles and immune parameters can plausibly affect chronic phase viremias, the blunted acute viremias seen in our PrEP breakthrough animals likely reflect continuous ARV drug activity since PrEP regimens were maintained for 15 to 20 weeks after infection. Here, we demonstrate that such reduced acute viremias are associated with limited virus diversity and little or no *env* diversification during the first 20 to 30 weeks of infection. In contrast to viruses from 4 out of 5 untreated macaques that acquired multiple *env* mutations in V1-V5, we only found two amino acid changes in the V5 region from 2 of the 4 PrEP failures. ARV treatment during acute infection may substantially limit replication and/or select for variants with reduced RT function relative to wild type. These virological findings are consistent with earlier immunological studies in macaques that noted delayed maturation of antibody avidity and reduced inflammatory responses and early CD4+ T cell loss associated with PrEP use or acute ARV treatment [6-10,18,19].

Our finding of limited viral evolution in the PrEP breakthrough animals may have important public health implications since early interactions between HIV and the host strongly influences pathogenesis and disease progression [20]. For instance, HIV-specific CD8+ T cell responses during primary infection are critical determinants for the subsequent control of viremia [21]. In HIV and SIV infections, the relatively homogeneous virus population seen at peak viremia subsequently undergoes significant evolution that rapidly increases the capacity to adapt to host immune responses [20]. A limited evolutionary potential associated with PrEP use may diminish the ability of HIV to escape host immune responses and enhance initial virus control. Limited virus evolution might also increase the window of vulnerability of HIV to vaccine-elicited immune responses as recently noted in vaccinated macaques receiving a sub-optimal dose of a topical microbicide [22].

The analysis of early *env* sequences from the five untreated macaques suggested that infection of these animals was initiated by a single founder virus, a finding that is consistent with the generally low multiplicity of infection associated with rectal infections in macaques and humans [5,14,23]. This observation is also consistent with the low (10 TCID_50_) virus inoculum used to infect these animals and the limited heterogeneity of the SHIV_162P3_ virus stock [14,15]. A similar analysis suggested that infection in our animals receiving PrEP was also initiated by a single variant. However, estimation of the number of transmitted/founder variants in these PrEP breakthrough animals is difficult since continuous ARV drug activity might potentially favor monospecific expansions of viruses with fitness advantages in the presence of ARVs. Selection of these viruses would likely shape the virus population in the animals.

The alterations in early viral and immunological parameters seen in this and earlier studies are noteworthy and deserve careful consideration. Blunted acute viremias might conceivably reduce gut CD4 T cell depletion, favor early restoration of mucosal T cells in the gut, and attenuate the course of infection [24]. However, our macaque model is not well suited to explore the long-term consequences of PrEP since we used a less pathogenic SHIV_162P3_ isolate. SHIV_162P3_ infections are characterized by early acute viremias similar to those seen in humans, but also low virus set points that generally result in a non-pathogenic disease course [25]. Unfortunately, the low set point viremias also preclude a more comprehensive analysis of viral evolution after PrEP discontinuation.

In summary, we show in macaques that drug selective pressures associated with ARV use for PrEP significantly limit early virus replication and evolution, reinforcing the need to evaluate these parameters in humans. The impact of PrEP on early infection in humans is not known and will likely depend on both the extent of ARV drug exposure at the time of infection and prior to HIV diagnoses. This interval will typically be several weeks and might be sufficient to affect early virus evolution and immune responses. Careful analysis of the characteristics of HIV infections in highly adherent participants from ongoing clinical trials may help to clarify how PrEP use may impact early infection, risks for secondary transmission, and the rate of progression to HIV disease.

## Conclusions

We explored in macaques the impact of PrEP on early viral dynamics and evolution. We demonstrate for the first time that PrEP significantly halts early virus evolution by containing the initial explosion of virus replication and diversification. These virological findings complement earlier immunological studies in macaques that noted altered immune responses associated with PrEP. Our findings emphasize the need to understand the characteristics of HIV infections that occur during PrEP implementation in humans.

## Methods

### Macaques and experimental SHIV infection

A total of nine male Indian rhesus macaques infected rectally with SHIV_162P3_ were studied. SHIV_162P3_ is a chimeric virus that contains the tat, rev, and env coding regions of HIV-1SF162 in a SIVmac239 background (National Institutes of Health AIDS Research and Reference Reagent Program [26]). All animals became infected during repeated weekly non-traumatic exposures to a low dose (10 TCID_50_) of SHIV_162P3_ as previously described [7,8]. Macaques AG80 and AG46 received daily PrEP with human-equivalent doses of FTC (20 mg/kg) and became infected after 5 and 10 rectal exposures, respectively. Macaques DK40 and DL6V received intermittent PrEP with 2 weekly human-equivalent doses of Truvada (22 mg/kg TDF and 20 mg/kg FTC) and became infected after 2 and 4 rectal challenges, respectively (6, 7). Macaques AM20, 17 V, AO86, AI22, and AG94 are untreated control animals that did not receive PrEP and became infected after 3, 1, 1, 5 and 3 rectal exposures, respectively [7,8] (Figure 1). The Institutional Animal Care and Use Committee of the Centers for Disease Control and Prevention approved these studies.

### Viral RNA extraction and cDNA generation

Viral RNA was extracted from longitudinal plasma specimens collected from macaques AG80, AG46, DK40, DL6V, AM20, 17 V, AO86, AG94 and AI22 at peak viremia and during 17 to 29 weeks of follow up using the QIAmp Viral RNA Mini Kit (Qiagen) (Figure 1). Specimens were selected based on detectable RNA in plasma by RT-PCR [7,8]. cDNA was generated by reverse transcription using primer QZR2 (5′ ACA GCT CCT AGC GTC ACT GCT C 3′) and Superscript III reverse transcriptase (RT) according to the manufacturer’s instructions (Invitrogen, Carlsbad, California). Briefly, 30 μl of plasma RNA were added to an RT cocktail containing 1.5 μl of primer QZR2 (10 μM), 3 μl dNTPs (10 mM), 3 μl DTT (100 mM), RNase OUT (120 units), and 600 units of Superscript III RT (Invitrogen) for a total 60 μl reaction. Reverse transcription was done at 50°C for 60 minutes and 55°C for 60 minutes. The mixture was heat inactivated at 70°C for 15 minutes and then treated with RNase H for 20 minutes at 37°C to remove the RNA template.

### Single genome amplification (SGA)

1.105 kb *env* sequences containing the V1-V5 region of gp120 (nucleotide positions 348 to 1,453 relative to HIV-1 isolate SF162, GenBank accession number EU123924) were generated by SGA. Briefly, serial dilutions of the RT-generated cDNA were made in 96-well plates to identify dilutions resulting in <30% of positive wells. Based on a Poisson distribution, amplifications at this dilution represent sequences derived from a single cDNA copy [11]. PCR reactions were done using 2 μl of cDNA at the appropriate dilution and a PCR cocktail containing primer QZF3 (5′ CCC ACA GAC CCT AAC CCA CAA GA 3′) and QZR2. The mixture was incubated for 2 minutes at 94°C followed by 40 cycles of 15 s at 94°C, 30s at 54°C, 90s at 68°C, and a final extension of 15 minutes at 68°C. A 2-μl volume was then used for a nested PCR reaction using primers QZF4 (5′ GCC ATG TGT AAA GTT AAC CCC ACT C 3′) and QZR3 (5′ GCC TTG GTG GGT GCT ACT CCT A 3′) and 45 cycles of 15 s at 94°C, 30 s at 54°C, 60 s at 68°C, with a final extension of 15 minutes at 68°C. Nested-PCR products were visualized using a pre-cast 96-well 1% agarose gel system and the E-Editor software tool (Invitrogen, Carlsbad, California). The number of positive wells was computed to ensure an amplification rate lower than 30%. Positive PCR products were then purified for subsequent sequence analysis using the Quick PCR purification kit (Qiagen).

### Sequence analysis

*Env* sequences were obtained using an ABI3130xl automated sequencer with primers QZR3, QZR5 (5′ GGT CCC CTC CTG AGG ATT GCT TA 3′), QZF7 (5′ CAG TCT AGC AGA AGA AGG GGT AG 3′), QZF5 (5′ CAC AAG CCT GTC CAA AGG TAT CC 3′, QZR6 (5′ GCT TTC CCC GGT CCT ATA GGT A 3′), and QZF8 (5′ TAC CTA TAG GAC CGG GGA AAG C 3′). PCR conditions included 25 cycles of 10s at 96°C and 10s at 55°C. The Vector NTI Advance program (version 11.5.0) was used to analyze the data and to infer amino acid sequences.

Phylogenetic analysis was done using the MEGA (version 5.0) software package. The Hasegawa, Kishino, and Yano model with gamma distributed rates and a proportion of invariable sites was inferred to be the best model of nucleotide substitution for *env* sequences from each individual animal, the SHIV inoculum, and all sequences combined by using maximum likelihood (ML) best fit analysis in the MEGA program. Nucleotide alignments were prepared using Clustal W in MEGA. All indels were manually removed from the alignments prior to phylogenetic analysis. Phylogenetic trees were inferred using neighbor joining (NJ) and ML methods and rooted with the SHIV_162P3_*env* consensus sequence. Viral diversity was inferred by analysis of neighbor-joining trees and the Highlighter tool available at http://www.hiv.lanl.gov/content/sequence/HIGHLIGHT/highlighter.html. The number of transmitted/founder variants that established infection was determined by exploring the pattern of *env* diversity at peak viremia. For this analysis, we excluded hyper mutated sequences and gaps, and assumed that variants with two or less unique nucleotide changes arise from the same transmitted founder virus [11]. Estimates of evolutionary divergence were done by pairwise analysis of individual sequences and the transmitted/founder virus using the maximum composite likelihood method in MEGA5, and results are expressed as number of nucleotide substitutions per site per year [27,28]. Mean nucleotide diversity and divergence for PrEP breakthrough animals and controls were compared by using a two-tailed t test (GraphPad Prism, version 5.04). Tropism was evaluated using the geno2pheno co-receptor tool (Version 2.0) available at http://coreceptor.bioinf.mpi-inf.mpg.de/index.php following the recommendations from the European Consensus Group. Potential N-linked glycosylation sites were analyzed using the N- GlycoSite tool available at http://www.hiv.lanl.gov/content/sequence/GLYCOSITE/glycosite.html.

## Abbreviations

PrEP, Pre-exposure prophylaxis; SGA, Single genome amplification; FTC, Emtricitabine; TDF, Tenofovir disoproxil fumarate.

## Competing interests

The authors declare that they have no competing interests.

## Authors’ contributions

QZ and SR carried out the molecular genetic studies and phylogenetic analysis. BS participated in the analysis and interpretation of data and helped to draft the manuscript. WH participated in the study design and helped to draft the manuscript. JGG-L conceived and coordinated the study and drafted the manuscript. All authors read and approved the final manuscript.
